# A Review of Ginseng Clinical Trials Registered in the WHO International Clinical Trials Registry Platform

**DOI:** 10.1155/2018/1843142

**Published:** 2018-02-06

**Authors:** Yingchun He, Juan Yang, Yinghua Lv, Junchao Chen, Fang Yin, Jihan Huang, Qingshan Zheng

**Affiliations:** Center for Drug Clinical Research, Shanghai University of Traditional Chinese Medicine, Shanghai, China

## Abstract

Although ginseng has long been broadly used in clinical settings around the world, few clinical trials on ginseng have been conducted. The objective of this study was to provide a comprehensive evaluation of the characteristics of ginseng clinical trials registered in the WHO International Clinical Trials Registry Platform (ICTRP) as of December 2017 regarding their frequency, design, type of ginseng, dosage, duration, condition, funding sources, and publication status. A total of 134 ginseng clinical studies were registered from 2002 to 2017, of which 60.4% were completed and 23.1% are actively recruiting participants. A large number of trials were associated with aspects of high-quality trial design. Overall, 94% of the trials employed randomized allocation to study arms, 78.4% were double-blind studies using placebo as one of the control groups, and 71% were published as completed trials. Trials whose sample size was restricted to fewer than 100 participants accounted for 74.7% of the total. Of the primary funding sources for ginseng studies, 67.2% were nonindustry organizations. The ginseng clinical trials were heterogeneous with respect to ginseng species and variety, indications, dose, duration, and participant characteristics. Clearly, stricter and methodologically suitable studies are needed to demonstrate the efficacy and safety of ginseng.

## 1. Introduction

Ginseng has been used for the treatment or prevention of diseases for thousands of years in eastern countries, and over the last three decades, it has gained popularity in the Americas, Canada, and Europe. Ginseng occupies a prominent position on the list of best-selling natural products in the world [1, 2]. In traditional Chinese medicine, ginseng is usually described as an “adaptogen,” a substance that can assist an organism in overcoming various types of stress and restore homeostasis [3]. It is included in the pharmacopoeias of China, Japan, Germany, France, Austria, and the United Kingdom. Asian ginseng (Panax ginseng Meyer) and American ginseng (Panax quinquefolius L.) are the two most recognized ginseng botanicals in the world [4]. Both Asian and American ginseng have a common mixture of active ingredients, primarily ginsenosides, in varying quantities, strengths, and ratios [5].

Although ginseng has long been broadly used in clinical settings worldwide, few clinical trials on ginseng have been conducted [6, 7]. Randomized controlled trials are the most stringent method of defining whether a cause-effect relation exists between treatment and result and of evaluating the benefits and risks of a treatment; this is also true for ginseng [8].

To our knowledge, no formal up-to-date assessment or inventory of the ginseng clinical trial landscape currently exists. With growing concern about how clinical research is performed, a comprehensive analysis of the portfolio of recent ginseng clinical research is both necessary and timely. In our study, we adopted systematic review methodology; supplementary 1 shows the information of the PRISMA Checklist.

The World Health Organization (WHO) established the International Clinical Trials Registry Platform (ICTRP) in 2005 to develop a platform for connecting clinical trial registries and providing a unified point of access to information on clinical trials conducted around the world. Over the last ten years, the ICTRP has grown into a platform that merges data from 17 different clinical trial registries, both national and regional [9].

The main objective of this study was to describe characteristics of the ginseng trials registered in the WHO ICTRP regarding their frequency, design, types of ginseng, dosage, duration, conditions, and funding sources. The secondary objective was to evaluate the publication status of the completed trials.

## 2. Materials and Methods

### 2.1. Data Source

We conducted a survey using the registration database of WHO registries through the ICTRP Search Portal (http://apps.who.int/trialsearch). The registries included the following: ClinicalTrials.gov, Australian New Zealand Clinical Trials Registry (ANZCTR), Brazilian Clinical Trials Registry (ReBec), Chinese Clinical Trial Register (ChiCTR), Clinical Research Information Service (CRiS), Cuban Public Registry of Clinical Trials (RPCEC), Clinical Trials Registry-India (CTRI), EU Clinical Trials Register (EU-CTR), German Clinical Trials Register (DRKS), ISRCTN, Iranian Registry of Clinical Trials (IRCT), Japan Primary Registries Network (JPRN), Pan African Clinical Trial Registry (PACTR), Republic of Korea (KCT), the Netherlands National Trial Register (NTR), Sri Lanka Clinical Trials Registry (SLCTR), and Thai Clinical Trials Registry (TCTR).

There were more than 300,000 trials on a wide array of illnesses and conditions registered in ICTRP as of 25 December 2017. These trials were performed all over the world [9].

### 2.2. Trial Selection

A query of the ICTRP Web site (http://apps.who.int/trialsearch/) was conducted by applying an advanced search function with “ginseng or ginsenoside” for “intervention.” No limitations were employed for the recruitment status, start and end time, condition, or country of recruitment.

To be eligible, a trial should have a description of using Panax ginseng or ginsenosides as a monopreparation as the intervention in the trial records. Trials for all indications were included. Trials were excluded if the intervention was not ginseng or if the trial employed a mixed intervention that included ginseng. The resulting records were manually screened for eligibility by two authors (HYC, JY). Discrepancies in the classification of records were resolved by discussion between the investigators.

### 2.3. Data Extraction

The following data for ginseng trial records were downloaded from the ICTRP database and imported into Excel on 25 December 2017: trial identifier, study title, primary sponsor, date of registration, register source, trial phase, recruitment status, anticipated enrolment sample size, condition studied, intervention, funding source, eligibility criteria (age group and sex), study design, and country/countries of recruitment.

Two investigators manually extracted descriptive information on the type, dosage, and duration of ginseng from the registered record in the source registry. We categorized the source of support as industry or nonindustry (university, hospital, and other) for analysis. If the lead sponsor was from industry or the study had at least one collaborator from industry, then the funding source was determined to be from industry. Otherwise, it was determined to be from nonindustry.

The publication status of complete trials was also extracted manually. Two investigators (HYC, JY) independently judged the publication status of each trial in December 2017 using a search protocol applied in previous research [10]. We manually searched Medline with the Trial identifier. If we did not identify a publication, we searched Medline again using the interventions, conditions studied and name of the principal investigator. The search results were refined, if needed, by specifying study design features, the name of the principal investigator (PI, when available), and the primary outcome. The Google Scholar database was searched in a similar fashion if the Medline search was unsuccessful. Differences were resolved by consensus.

### 2.4. Statistical Analyses

Descriptive statistics were used to characterize the ginseng trials extracted from the ICTRP Search Portal. Continuous variables were reported as medians and interquartile ranges, and categorical variables were reported as proportions. All statistical analyses were performed using the SAS software (version 9.3; SAS Institute Inc., Cary, NC, USA). Missing values were excluded from analysis unless indicated.

## 3. Results

### 3.1. Basic Trial Characteristics

**Figure 1:**
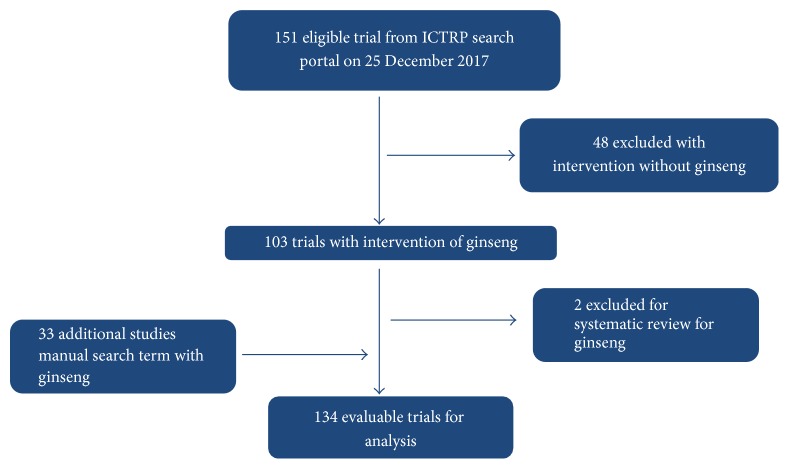
Flowchart of trial selection.

A total of 151 registration records were retrieved from the registries. Of these trials, 50 were excluded from further analysis because the intervention was not ginseng (48 trials) or the records were systematic review for ginseng (2 trials). 33 additional studies were found by text searching with relevant terms. As a result, 134 records were defined as ginseng clinical trials (GCTs) (Figure 1).

**Figure 2:**
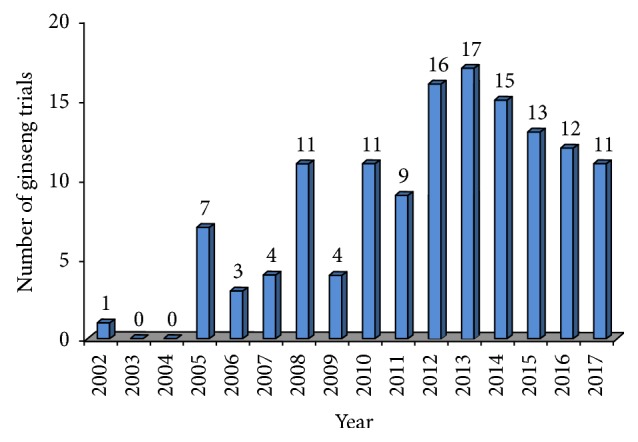
Number of registration of ginseng clinical trials from 2002 to 2017.

All 134 of the GCTs were registered during the period from 2002 to 2017. The number of registrations increased from 1 in 2002 to a peak of 17 in 2013 (Figure 2).

Ginseng trials were found in the following 8 registries: ANZCTR (8), ChiCTR (9), clinicalTrials.gov (85), EU-CTR (1), IRCT (10), ISRCTN (1), JPRN (7), and KCT (13). No registration records were found in CTRI, DRKS, NTR, PACTR, ReBec, RPECE, SLCTR, or TCTR.

A complete summary of the basic characteristics of all evaluated trials can be found in Table 1. With respect to recruitment status, 81 (60.4%) have been completed, 31 (23.1%) are actively recruiting subjects, and most of the remaining trials have not yet begun to recruit participants. The primary purpose in 71 (53%) GCTs was treatment disease, followed by supportive care (25, 18.7%) prevention (19, 14.2%) and basic science studies (16, 11.9%). Safety/efficacy was the primary endpoint in 80 (59.7%) trials, followed by efficacy (36, 26.9%) and pharmacokinetics or pharmacodynamics (8, 6.6%). Allocation to intervention arms via randomization was dominant (126, 94.0%) among the ginseng trials. Parallel group assignment was the most common allocation scheme (102, 76.1%), followed by crossover assignment (24, 17.9%), single group assignment (7, 5.2%), and factorial assignment (1, 0.7%). 105 (78.4%) studies used placebo as one of the control groups. A majority (105, 78.4%) of trials reported double blinding and 9% (12) single blinding, and 11.2% (15) used an open label design. The median of anticipated enrolment sample size was 60 subjects (interquartile range 39–100 subjects). Studies whose sample size was restricted to fewer than 100 participants accounted for 74.7% of the trials (100). Overall, 130 (97%) of the ginseng trials included adults, and 76 (56.7%) of the ginseng trials included seniors. Only 7 (5.2%) enrolled children.

Phase 0 to II trials were the most common (49; 36.6%), and approximately one-third of the studies were Phases III to IV (39; 29.1%). The remainder of the studies did not specify a phase (46, 34.3%).

Overall, the primary funding sources of the ginseng studies were nonindustry (90, 67.2%). Only 44 trials (32.8%) were funded by industry sources.

### 3.2. Ginseng for Intervention

Of the 134 trials analyzed, 91 (71.6%) were conducted using Panax ginseng as the intervention of interest, followed by American ginseng (22, 16.4%) and ginsenoside (18, 13.4%). Three (2.2%) studies included two different types of P. ginseng. In 75 (56%) trials, ginseng was given as capsules. In 15 (11.2%) trials, ginseng was given as tablets, 5 (3.7%) trials used oral liquid, and 1 (0.7%) used tea. Thirty-six (26.9%) trials did not show what preparation was used. The dosages administered daily differed in line with the type of ginseng. Panax ginseng administration was from 0.2 g to 9 g every day. American ginseng administration was from 0.1 g to 15 g, whereas ginsenoside intake was from 0.01 g to 3 g per day. Sixteen trials were involved in comparing dose dependency. The period of ginseng administration was 3–14 days for the short-term trials (19 trials), and the longest period was 52 weeks (1 trials). Approximately 59% (79) of the trials had an intake of 4 to 12 weeks. There were 21 trials with a 4-week intake, 24 trials with an 8-week intake, 29 trials with a 12-week intake, and 7 trials with a 24-week intake. Seven trials were single-time dosing. Seventeen trials did not state the duration of ginseng intake.

### 3.3. Information on Conditions

The information on the target conditions was as follows. Twenty-four (17.9%) trials recruited healthy subjects. The most common conditions were diabetes and metabolic syndrome (23, 17.2%). Fatigue (19, 14.2%), cognitive disorders (9, 6.7%), erectile dysfunction (11, 8.2%), hypertension (9, 6.7%), cancer related symptoms (7, 5.2%), respiratory tract infection (7, 5.2%), and mental disorders (5, 3.7%) were of special concern. The rest of the studies examined the use of ginseng among people with one of the following 12 conditions: chronic obstructive pulmonary disease (3, 2.2%), obesity (2, 1.5%), ischemic stroke (3, 2.2%), allergic rhinitis (2, 1.5%), postmenopausal status (2, 1.5%), dry mouth (1, 0.7%), cold hypersensitivity (1, 0.7%), noise-induced hearing loss (1, 0.7%), brucellosis (1, 0.7%), chronic stable angina pectoris (1, 0.7%), rheumatoid arthritis (1, 0.7%), detoxification of bisphenol A (1, 0.7%), and liver function (1, 0.7%).

### 3.4. Geographic Distribution

Almost all of the registered ginseng trials were conducted exclusively domestically, except for 2 trials (NCT00401089, NCT00240461). More than half of the trials took place at a single site (74, 55.2%), and 16 (11.9%) trials were multicenter studies. Forty-two (31.3%) trials did not state site information. The majority of the trials are in Korea (51, 34.2%), Canada (19, 15.3%), China (18, 15.3%), and US (15, 13.5%); however, Iran (11, 6.3%), Japan (7, 3.6%), and Australia (4, 3.6%) are also involved in several ginseng trials. One trial was conducted in each of the following countries: Brazil, Denmark, Spain, Sweden, Malaysia, South Africa, Italy, and Egypt.

### 3.5. Publication

A total of 72 GCTs were listed as “completed” as of December 2014. We found that 71% (*n* = 51) of the trials had published their results in peer-reviewed biomedical journals and 29% (*n* = 21) remained unpublished after we manually searched the Medline and Google Scholar databases.

## 4. Discussion

By using the ICTRP Search Portal, we completed the first comprehensive examination of recent ginseng clinical research. From this analysis we demonstrated that GCTs make up only small fraction of all registered clinical trials. Furthermore, we determined that the majority of ginseng trials are of limited sample size, they are single-center trials with high heterogeneity in interventions and conditions, and many are not funded by industry.

Although other studies have evaluated published ginseng clinical trials [11–20], none have focused exclusively on registered clinical trials of ginseng. Our goal in performing this review was to determine the current trends in GCT research and identify areas for potential improvement or future prioritization. Accordingly, we believe that our results offer some intriguing insights into the ginseng clinical trial domain.

The total number of registered ginseng clinical trials has been increasing steadily over recent years, particularly in Korea and China. The world ginseng annual sales exceeded $2,000 million in 2014, with the continuous global growth of healthy food consumption [21]. However, too few studies are being conducted on ginseng. The efficacy and safety of ginseng must be explicitly addressed, and ginseng clinical research mush be supported.

Although ginseng studies only accounted for a small fraction of all registered trials in the ICTRP Search Portal, the majority of the trials involved elements of good quality trial design. More than 90% of the ginseng trials applied a randomization procedure, and three-quarters were double-blinded. The majority of the trials included a primary safety/efficacy endpoint. These values are higher than the average of all 40,970 interventional trials registered in ClinicalTrials.gov from 2007 to March of 2010 [22].

In our review, we found a randomized, double-blind, placebo-controlled, multicenter trial (NCT02428998), and its purpose was to evaluate the safety after 24 weeks of ginseng intake. The target sample size was 1000 adults. This is an encouraging finding, and it is our hope that an increasing proportion of future trial designs will incorporate evaluations of safety-related longer-term outcomes with large samples because the great majority of ginseng trials are of relatively short duration at the present time.

Another positive finding is that a comparatively large proportion of finished GCTs have already been published in peer-reviewed biomedical journals. Our observed publication rate (71%) of registered GCTs is higher than the rates observed in other clinical trials [11, 23–26]. Reasons for failure to publish include lack of positive results, time restriction, limited resources, change of interests, or simply failure to have the paper accepted by a journal [26]. Publication bias may cause a waste of resources due to a needless repetition of trials and the possibility of harm to the study subjects [27]. Therefore, we need to make further efforts to promote accountability among researchers and industry sponsors to facilitate the timely publication of completed trials.

Fully 70% of ginseng studies registered from 2002 to 2015 included fewer than 100 participants by design. This perhaps is due to early-phase trials occupying a greater proportion with more Phases 0 to II trials compared with Phases III to IV trials. Small sample size studies are proper in some situations (e.g., early-phase clinical study, trial of rare/orphan diseases). However, small-scale trials are not as informative in many other cases, such as establishing the effectiveness of treatments with modest effects and comparing effective treatments to enable better decisions in practice [28, 29]. Small, low statistical power studies have a high risk of type II error, that is, failing to reject the null hypothesis and inappropriately concluding that a therapy or intervention is ineffective [30].

The present study is similar to many studies done previously [15, 17, 18] showing that GCTs have been heterogeneous in terms of ginseng species or variety, indications, dose, duration, and participant characteristics. Most features of these trials were diverse. For example, the subjects were either healthy volunteers or people with health conditions such as diabetes, fatigue, hypertension, stroke, or mental disorders. Even after classifying the trials by disease/condition subcategories, the diversity remained. The duration of ginseng intake varied from 4 to 24 weeks, and the P. ginseng doses also differed, ranging from 0.3 to 15 g every day for the diabetes trials. The heterogeneity of ginseng trials makes the study results inconsistent and often difficult to interpret because the use of P. ginseng may differ. This also made it difficult to compare trials and judge whether their proposals for dose and duration are suitable.

Consistency in chemical composition and pharmacological properties is fundamental for safety and the effective intake of herbal drugs, but ginseng and other herbal products frequently fail to meet this standard [31]. The ginsenoside content of ginseng can vary depending on the genetic variability, age, locality, preservation method, harvest season, extraction method, and other environmental factors [32, 33]. Quality control and standardization of active ingredients must therefore be made available for the further elucidation of the efficacy/safety in ginseng clinical trials.

This study has several limitations. Our analysis includes only registered GCTs, and some GCTs may thus have been missed as a result of not being registered. In addition, this analysis of the ICTRP registry likely underestimates the prevalence of ginseng studies because many countries do not have a legal requirement for clinical trial registration (e.g., China, Japan) [34], although many investigators from these countries use ICTRP to satisfy ICMJE registration requirements. Moreover, the accuracy, validity, and completeness of the data entered in the portal are solely dependent on self‐reporting by the study sponsors and investigators. Thus, we cannot guarantee that our results are absolutely accurate. Nonetheless, we believe that we have provided one of the largest and most complete surveys of ginseng clinical trials to date. Another limitation of our present study is that efficacy and safety issues are not included. However, the literatures have reported that ginseng generally has a good safety profile and efficacy is also evaluated [15, 18, 35]. In future research, we will pay more attention to the safety and effectiveness of ginseng clinical trials.

## 5. Conclusion

In spite of the worldwide expansion of ginseng products as complementary and alternative medicine, P. ginseng has only been studied in a limited number of clinical trials with a relatively small number of subjects and with various conditions ranging from healthy subjects to patients with symptoms. Clearly, stricter and methodologically thorough research is needed to demonstrate the efficacy and safety of ginseng.

## Figures and Tables

**Table 1:** General characteristics of registered ginseng trials.

Characteristic	Category	Total (*N* = 134) [*n* (%)]
Recruitment status	Not yet recruiting	11 (8.2)
	Recruiting	31 (23.1)
	Completed	81 (60.4)
	Enrolling by invitation	1 (0.7)
	Active, not recruiting	7 (5.2)
	Available	2 (1.5)
	Suspended	1 (0.7)
Phase	Phase 0	2 (1.5)
	Phase 1	11 (8.2)
	Phase 1∣Phase 2	7 (5.2)
	Phase 2	29 (21.6)
	Phase 2∣Phase 3	7 (5.2)
	Phase 3	22 (16.4)
	Phase 4	10 (7.5)
	Unknown/missing	46 (34.3)
Endpoint classification	Safety/efficacy	80 (59.7)
	Safety	9 (6.7)
	Efficacy	36 (26.9)
	Pharmacokinetics	5 (3.7)
	Pharmacodynamics	1 (0.7)
	Bioavailability	3 (2.2)
Primary purpose	Treatment	71 (53)
	Supportive care	25 (18.7)
	Prevention	19 (14.2)
	Basic science	16 (11.9)
	Unknown/missing	3 (2.2)
Gender	Both	106 (79.1)
	Male	19 (14.2)
	Female	9 (6.7)
Age group_ _^*∗*^	Child	4 (3.0)
	Child∣adult	2 (1.5)
	Child∣adult∣senior	1 (0.7)
	Adult	52 (38.8)
	Adult∣senior	75 (56.0)
Funding source	Industry	44 (32.8)
	Nonindustry	90 (67.2)
Intervention model	Parallel assignment	102 (76.1)
	Crossover assignment	24 (17.9)
	Factorial assignment	1 (0.7)
	Single group assignment	7 (5.2)
Allocation	Randomized	126 (94.0)
	Nonrandomized	8 (6.0)
Masking	Double-blind	105 (78.4)
	Single-blind	12 (9)
	Open label	15 (11.2)
	Unknown/missing	2 (1.5)
Number of arms	1	5 (3.7)
	2	96 (71.6)
	3	22 (16.4)
	4	8 (6)
	≥5	3 (2.2)
Placebo comparator	Yes	105 (78.4)
	No	29 (21.6)
Expected sample size	Median (25%,75%)	60 (39.5, 100)
	0 to 50	49 (36.6)
	51 to 100	51 (38.1)
	101 to 200	18 (13.4)
	201 to 500	12 (9)
	501 to 1000	3 (2.2)

^*∗*^Adults: age is 18~65, seniors: age > 65, and children: age < 18.

## Supplementary Materials

Supplementary Materials Supplement 1 shows the information of the PRISMA 2009 Checklist.

## References

[1] FM P. (2009). Panax ginseng. Monograph. *Alternative Medicine Review*.

[2] FC L. (1992). *Facts about ginseng: the elixir of life*.

[3] Brekhman II., Dardymov IV. (1969). New substances of plant origin which increase nonspecific resistance. *Annual Review of Pharmacology*.

[4] Ang-Lee M. K., Moss J., Yuan C. S. (2001). Herbal medicines and perioperative care. *Jama*.

[5] Cui J.-F. (1995). Identification and quantification of ginsenosides in various commercial ginseng preparations. *European Journal of Pharmaceutical Sciences*.

[6] Firenzuoli F., Gori L. (2007). Herbal medicine today: clinical and research issues. *Evidence-Based Complementary and Alternative Medicine*.

[7] Chen S., Wang Z., Huang Y. (2014). Ginseng and anticancer drug combination to improve cancer chemotherapy: a critical review. *Evidence-Based Complementary and Alternative Medicine*.

[8] Sibbald B., Roland M. (1998). Understanding controlled trials: Why are randomised controlled trials important?. *BMJ*.

[9] WHO International Clinical Trials Registry Platform. http://apps.who.int/trialsearch/.

[10] Khan N. A., Singh M., Spencer H. J., Torralba K. D. (2014). Randomized controlled trials of rheumatoid arthritis registered at clinicaltrials.gov: What gets published and when. *Arthritis & Rheumatology*.

[11] Vogler B. K., Pittler M. H., Ernst E. (1999). The efficacy of ginseng. A systematic review of randomised clinical trials. *European Journal of Clinical Pharmacology*.

[12] Jang D.-J., Lee M. S., Shin B.-C., Lee Y.-C., Ernst E. (2008). Red ginseng for treating erectile dysfunction: a systematic review. *British Journal of Clinical Pharmacology*.

[13] Soo-Jin P., Yang-Jin C., Jae-Ho P., Hee-Do H. (2006). Meta-analysis of studies and patents on Korean ginseng in recent 5 years in Korea and prospective needs. *Journal of Ginseng Research*.

[14] Buettner C., Yeh G. Y., Phillips R. S., Mittleman M. A., Kaptchuk T. J. (2006). Systematic review of the of ginseng on cardiovascular risk factors. *Annals of Pharmacotherapy*.

[15] Choi J., Kim TH., Choi TY., Lee MS. (2013). Ginseng for health care: a systematic review of randomized controlled trials in Korean literature. *PLoS One*.

[16] Lee M. S., Yang E. J., Kim J.-I., Ernst E. (2009). Ginseng for cognitive function in alzheimer's disease: A systematic review. *Journal of Alzheimer's Disease*.

[17] Shergis J. L., Zhang A. L., Zhou W., Xue C. C. (2013). Panax ginseng in Randomised controlled trials: A systematic review. *Phytotherapy Research*.

[18] Lee N.-H., Son C.-G. (2011). Systematic review of randomized controlled trials evaluating the efficacy and safety of ginseng. *JAMS Journal of Acupuncture and Meridian Studies*.

[19] Young-Sook K., Jung-Yoon W., Chang-Kyun H., Il-Moo C. (2015). Safety analysis of panax ginseng in randomized clinical trials: a systematic review. *Medicines*.

[20] Seida J. K., Durec T., Kuhle S. (2011). North american (panax quinquefolius) and asian ginseng (panax ginseng) preparations for prevention of the common cold in healthy adults: a systematic review. *Evidence-Based Complementary and Alternative Medicine*.

[21] Baeg I.-H., So S.-H. (2013). The world ginseng market and the ginseng (Korea). *Journal of Ginseng Research*.

[22] Califf R. M., Zarin D. A., Kramer J. M. (2012). Characteristics of clinical trials registered in ClinicalTrials.gov, 2007-2010. *Jama*.

[23] Stockmann C., Sherwin C. M. T., Koren G. (2014). Characteristics and publication patterns of obstetric studies registered in ClinicalTrials.gov. *Clinical Pharmacology and Therapeutics*.

[24] Gandhi R., Jan M., Smith H. N., Mahomed N. N., Bhandari M. (2011). Comparison of published orthopaedic trauma trials following registration in Clinicaltrials.gov. *BMC Musculoskeletal Disorders*.

[25] Wildt S., Krag A., Gluud L. (2011). Characteristics of randomised trials on diseases in the digestive system registered in ClinicalTrials.gov: A retrospective analysis. *BMJ Open*.

[26] Ross J. S., Tse T., Zarin D. A., Xu H., Zhou L., Krumholz H. M. (2012). Publication of NIH funded trials registered in ClinicalTrials.gov: Cross sectional analysis. *BMJ*.

[27] McCray A. T., Ide N. C. (2000). Design and implementation of a national clinical trials registry. *Journal of the American Medical Informatics Association *.

[28] Yusuf S., Collins R., Peto R. (1984). Why do we need some large, simple randomized trials?. *Statistics in Medicine*.

[29] Califf R. M., DeMets D. L. (2002). Principles from clinical trials relevant to clinical practice: Part I. *Circulation*.

[30] PD E. (2010). *The essential guide to effect sizes: Statistical power, meta-analysis, and the interpretation of research results*.

[31] Wahid F., Khan T., Subhan F., Khan M. A., Kim Y. Y. (2010). Ginseng pharmacology: Multiple molecular targets and recent clinical trials. *Drugs of the Future*.

[32] Liberti L. E., Marderosian A. D. (1978). Evaluation of commercial ginseng products. *Journal of Pharmaceutical Sciences*.

[33] JD P., LA A. (1984). Ginseng, quality, safety and efficiency. *Pharmaceutical Journal*.

[34] Ogino D., Takahashi K., Sato H. (2014). Characteristics of clinical trial websites: information distribution between ClinicalTrials.gov and 13 primary registries in the WHO registry network. *Trials*.

[35] Kim Y. S., Woo J. Y., Han C. K., Chang I. M. (2015). Safety analysis of panax ginseng in randomized clinical trials: a systematic review. *Medicines (Basel)*.

